# External Doses Available for Epidemiological Studies Related to the Fukushima Health Management Survey: First 4-month Individual Doses and Municipality-average Doses for the First Year

**DOI:** 10.2188/jea.JE20210166

**Published:** 2022-12-05

**Authors:** Tetsuo Ishikawa, Seiji Yasumura, Keiichi Akahane, Shunsuke Yonai, Akira Sakai, Osamu Kurihara, Mitsuaki Hosoya, Ritsu Sakata, Tetsuya Ohira, Hitoshi Ohto, Kenji Kamiya

**Affiliations:** 1 Radiation Medical Science Center for the Fukushima Health Management Survey, Fukushima Medical University, Fukushima, Japan; 2 National Institutes for Quantum Science and Technology, Chiba, Japan; 3 Radiation Effects Research Foundation, Hiroshima, Japan; 4 Research Institute for Radiation Biology and Medicine, Hiroshima University, Hiroshima, Japan

**Keywords:** Fukushima accident, external dose, first year

## Abstract

**Background:**

One of the components of the Fukushima Health Management Survey (FHMS) is the Basic Survey, which estimates individual external doses for the first 4 months after the 2011 nuclear power plant accident. However, external exposure continues long-term. According to estimations by international organizations, the external dose during the first year accounts for a significant part of the long-term dose. Thus, the present study was intended to estimate the first-year doses by extrapolating the Basic Survey results.

**Methods:**

For most municipalities of non-evacuated areas, ambient dose rate had been continuously measured for at least one designated point in each municipality after the accident. In the present study, a municipality-average dose received by residents for a period was assumed to be proportional to the ambient dose measured at the designated point of that municipality during the same period. Based on this assumption, 4-month municipality-average doses calculated from the Basic Survey results were extrapolated to obtain first-year doses.

**Results:**

The extrapolated first-year doses for 49 municipalities in the non-evacuated areas had a good correlation with those estimated by UNSCEAR, although the extrapolated doses were generally higher (slope of the regression line: 1.23). The extrapolated municipality-average doses were in reasonable agreement (within 30%) with personal dosimeter measurements, suggesting that the extrapolation was reasonable.

**Conclusion:**

The present paper reports the first 4-month average doses for all 59 municipalities of Fukushima Prefecture and the extrapolated first-year doses for 49 municipalities. The extrapolated doses will be the basis for future epidemiological studies related to the FHMS.

## INTRODUCTION

The UNSCEAR 2013 report summarized radiation doses to the public and workers due to the Fukushima Daiichi Nuclear Power Plant (FDNPP) accident.^1^ Based on the dose assessment, the UNSCEAR Committee reported that no discernible increased incidences of radiation-related health effects were expected among exposed members of the public or their descendants. However, there is ongoing debate in Japan as to whether health effects observed after the FDNPP accident are radiation-induced or not.^2^^,^^3^ In particular, thyroid cancer is a concern.^4^^–^^6^

One component of the Fukushima Health Management Survey (FHMS) is the Basic Survey, which estimates individual external doses for the surveyed population.^7^^,^^8^ The estimated external doses are linked with results from other surveys within the FHMS, such as thyroid ultrasound examination, in a database. The database has been used for analysis between the doses and the survey results.^6^^,^^9^

Although the Basic Survey estimates individual doses for the first 4 months after the accident, external exposure continues long-term. According to dose estimations by international organizations, the first-year dose accounts for a significant part of long-term dose. The World Health Organization^10^^–^^12^ considered it reasonable to assume that the ratio of long-term dose to the first-year dose would be equal to 2. UNSCEAR^1^ estimated the ratio of 10-year dose to the first-year dose was around 2.2 to 2.4 for non-evacuated areas, and that of lifetime dose to the first-year dose was 3.3. This meant that around 40% of the 10-year dose was given in the first year.

In this study, an extrapolation method for estimating first-year municipality-average doses from the Basic Survey data (4-month individual doses) was presented. The first-year doses obtained by the method were compared with UNSCEAR estimations. Recently, the UNSCEAR 2020 report^13^ was published (March 2021). Although it shows ranges (minimum and maximum) of first-year municipality-average effective doses for non-evacuated areas of Fukushima Prefecture, each municipality-average dose is not shown. These will be given in attachments of the 2020 report, which will be published later. Thus, municipality-average dose values given in the UNSCEAR 2013 report were used for the comparison. In addition, average external doses for some municipalities during periods within the first year were estimated by the method. They were compared with doses measured by personal dosimeter measurements to validate the extrapolation method.

## METHODS

### Basic Survey outline

Details of the Basic Survey are given elsewhere.^14^ Briefly, it is a self-administered questionnaire survey that asked subjects to record and send back information on their behavior (including time spent indoors and outdoors and time of moves) in the first 4 months after the accident start date (March 11, 2011). The target population of the Basic Survey has been people who were registered residents of Fukushima Prefecture from March 11 to July 1, 2011. The respondents’ behavior records were digitalized, and a computer program calculated individual effective doses due to external exposure by superimposing the behavior records with daily ambient dose equivalent rate maps.^15^

The individual doses for the first 4 months were calculated for 465,999 questionnaire respondents as of March 31, 2019.^16^ Although the response rate for the whole target population was around 28%, the dose distribution obtained represents the dose distribution for all of Fukushima Prefecture.^17^ The individual doses were classified by their residential municipalities at the time of the accident.

### Characteristics of individual doses estimated using the Basic Survey

**Figure 1.**
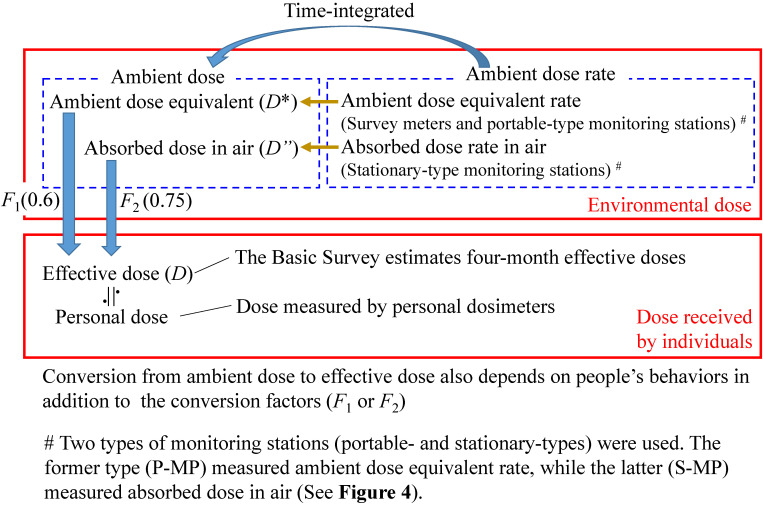
Technical terms related to dose used in the present paper

**Figure 2.**
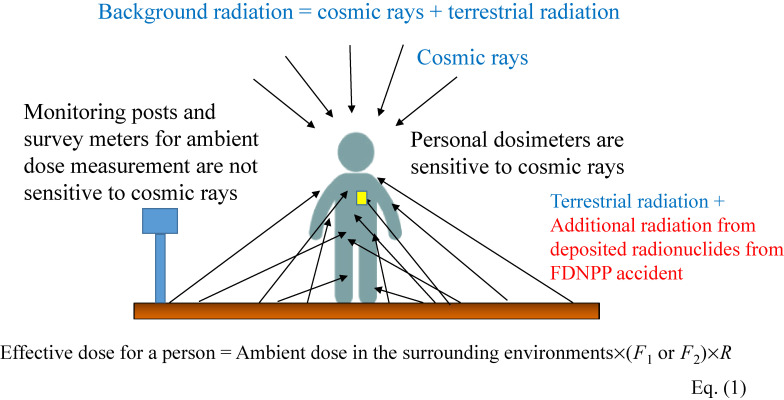
Background radiation sources and difference in sensitivity to these sources by measurement devices

The Basic Survey estimates effective doses due to external exposure resulting from the accident. On the other hand, all human beings are exposed to natural radiation and its annual average effective dose in Japan is estimated to be 2.2 mSv, including internal exposure.^18^ Regarding external exposure from natural radiation, there are two main sources: cosmic rays and terrestrial radiation. Hereafter, the dose from these two sources is called “background dose” and the dose due to the accident is called “additional dose”. Technical terms related to dose used in the present paper are explained in Figure 1 and Figure 2.

According to a previous analysis of the Basic Survey data, the dependence of individual doses on age group (adults, children, and infants) and sex was relatively small (less than 10% for non-evacuated areas).^19^ Thus, the individual doses were analyzed without dividing by age and sex.

The Basic Survey has an important aspect of being a health service to residents by providing dose estimates from individuals who provided their behaviors in the questionnaires and submitted them. Considering the uncertainty of individual doses estimated by the Basic Survey, it was decided that the dose estimate for residents should be rounded to the first decimal place for doses of less than 10 mSv and to the ones place for doses exceeding 10 mSv. It was also decided that these rounded dose estimates for residents should be available for research purposes, because the same information should be fairly provided to both researchers and residents; hence, they are stored in a database which is open to researchers. For doses less than 0.1 mSv, they are stored as “less than 0.1 mSv” without any numerical data. Data used in this study were extracted from the database. A certain numerical value should be assigned to calculate arithmetic means. Here, 0.05 mSv was assigned for “less than 0.1 mSv”.

### Method for estimating the first-year dose for each municipality

The Basic Survey questionnaire form was designed to fill in behavior records in the first 4 months after the accident. In the questionnaire, respondents were not asked to give information on behaviors after that. Also, there were no surveys which obtained people’s behavior records after the first 4 months. Thus, it is not possible to estimate individual doses by applying the same method as the Basic Survey for the subsequent period of the following 8 months. Personal dosimeter measurements were started in Fukushima Prefecture several months after the accident, but measurement periods differed among municipalities and did not cover all these 8 months.

On the other hand, ambient dose rate was continuously measured for at least one designated point in most non-evacuated area municipalities in the first year.^20^ Hereafter, “ambient dose” is used to refer to ambient dose equivalent or absorbed dose in air or both (see Figure 1). Thus, the present study was intended to estimate an average effective dose for a population of each municipality in the subsequent 8 months using the ambient dose rate data on the basis of the following ideas.

A relationship between additional effective dose for a reference person for a certain period, *D*(*t*), and additional ambient dose equivalent in the person’s surrounding environment for the same period $D^{*}(t)$ can be related as follows^21^:

$$D(t)=D^{*}(t)\times F_{1}\times R$$ (1)

*F*_1_ is the conversion coefficient from the ambient dose equivalent to effective dose (Figure 1). For the situations in Fukushima Prefecture, it was estimated to be around 0.6.^22^

*R* represents a radiation reduction factor, and it depends on the daily indoor and outdoor time budget and shielding effects of the building where the person stayed indoors. The government’s dose estimation model employs a radiation reduction coefficient (*R*_G_) of 0.6 under the assumption that per day people spend 8 h outside and 16 h inside in a building with a 0.4 shielding effect^21^:

$$R_{G}=\frac{\left(1\times 8+0.4\times 16\right)}{24}=0.6$$ (2)

**Figure 3.**
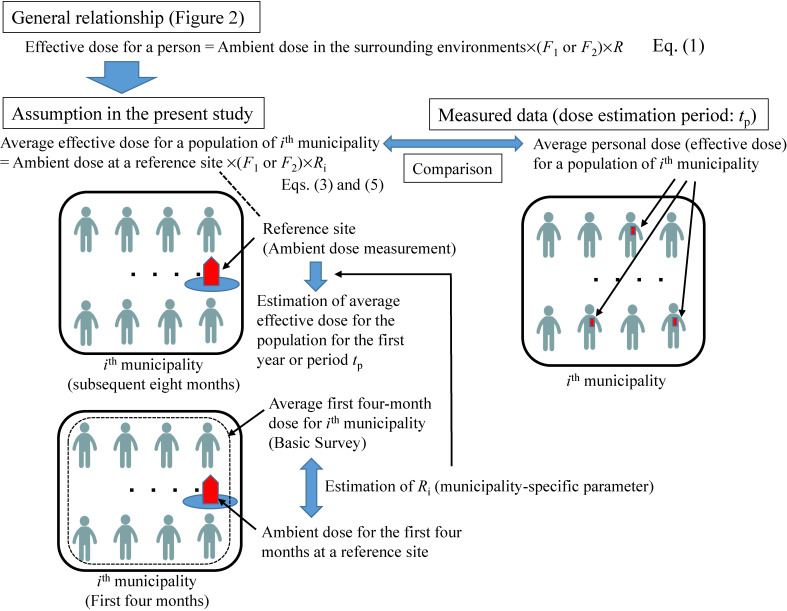
Scheme of the method for estimating first-year dose and its validation

In this study, equation (1) was assumed applicable to the population in each non-evacuated area municipality (Figure 3). On this assumption, an average additional effective dose during period *t* (less than or equal to 1 year) for the population of *i*^th^ municipality *D_i_*(*t*) is estimated by:

$$D_{i}(t)=D_{i}^{*}(t)\times F_{1}\times R_{i}$$ (3)

where $D_{i}^{*}(t)$ is the additional ambient dose equivalent measured for the same period at a reference site in *i*^th^ municipality, *R*_i_ is an average radiation reduction factor for *i*^th^ municipality. Most variables used in equations (3) to (10) are summarized in Table 1.

**Table 1.** Explanation of variables used in the present paper

Measurement devices			Not applicable (Estimation by calculation)	Stationary-type monitoring posts	Portable-type monitoring posts	Survey meters	Personal dosimeters	

Estimated types of dose			Effective dose	Absorbed dose rate in air	Ambient dose equivalent	Ambient dose equivalent	Personal dose	No. of Equation where variables used

Basic symbol			*D*	*D*′′	$D^{*}$	$D^{*}$	*D^p^*	

	Place of interest	Period for dose estimation						
Measured dose including background	*i*^th^ municipality	*t*_p_	—	—	$D_{Mi}^{*}(t_{p})$	$D_{Mi}^{*}(t_{p})$	$D_{Mi}^{p}(t_{p})$	(9), (10)
		*T*	—	—	—	$D_{Mi}^{*}(T)$	—	(6), (7)

	*R*^th^ region	*T*	—	$D_{MR}^{\prime \prime }(T)$	$D_{MR}^{*}(T)$	—	—	(6), (7)
		First 4 months	—	$D_{MR}^{\prime \prime }(4m)$	$D_{MR}^{*}(4m)$	—	—	(6), (7)

Additional dose due to the accident	*i*^th^ municipality	*t*	*D_i_*(*t*)	$D_{i}^{\prime \prime }(t)$	$D_{i}^{*}(t)$	$D_{i}^{*}(t)$	—	(3), (5)
		*T*		—	—	$D_{i}^{*}(T)$		(6), (7)
		*t*_p_	*D_i_*(*t_p_*)	—	$D_{i}^{*}(t_{p})$	$D_{i}^{*}(t_{p})$	$D_{i}^{p}(t_{p})$	(8), (9)
		First 4 months	*D_i_*(4*m*)	$D_{i}^{\prime \prime }(4m)$	$D_{i}^{*}(4m)$	$D_{i}^{*}(4m)$	—	(4), (6), (7)
		Subsequent 8 months	—	$D_{i}^{\prime \prime }(8m)$	$D_{i}^{*}(8m)$	$D_{i}^{*}(8m)$	—	(4)
		First year	*D_i_*(1*y*)	$D_{i}^{\prime \prime }(1y)$	$D_{i}^{*}(1y)$	$D_{i}^{*}(1y)$	—	(4)

	*R*^th^ region	T	—	$D_{R}^{\prime \prime }(T)$	$D_{R}^{*}(T)$	—	—	(6), (7)
		First 4 months	—	$D_{R}^{\prime \prime }(4m)$	$D_{R}^{*}(4m)$	—	—	(6), (7)

Background dose due to terrestrial radiation	*i*^th^ municipality	*T*	—	—	$D_{Bi}^{*}(T)$	$D_{Bi}^{*}(T)$	—	(6), (7)
		*t*_p_	*D_Bi_*(*t_p_*)	—	$D_{Bi}^{*}(t_{p})$	$D_{Bi}^{*}(t_{p})$	$D_{Bi}^{p}(t_{p})$	(9), (10)

	*R*^th^ region	*T*	—	$D_{BR}^{\prime \prime }(T)$	$D_{BR}^{*}(T)$	—	—	(6)
		First 4 months	—	$D_{BR}^{\prime \prime }(4m)$	$D_{BR}^{*}(4m)$	—	—	(6)

Background dose due to cosmic rays	all municipalities	*t*_p_	*D_UB_*(*t_p_*)	No sensitivity	No sensitivity	No sensitivity	$D_{Bi}^{p}(t_{p})$	(9), (10)

The assumption was based on: (1) no drastic changes in personal behavior patterns between the first 4 months and subsequent 8 months after the accident; and (2) most people continued to live in the same living environments where ambient dose rates were decreasing in the same trend as the reference site. For areas where the number of migrating people was small compared with the total target municipality population (ie, non-evacuated areas), this assumption was reasonable.

Then, a municipality-average additional dose for the first year can be estimated from municipality-average doses estimated using the Basic Survey *D_i_*(4*m*) by:

$$\begin{array}{rl} D_{i}(1y) & =D_{i}^{*}(1y)\times F_{1}\times R_{i}=(D_{i}^{*}(4m)+D_{i}^{*}(8m))\times F_{1}\times R_{i}\\ & =D_{i}(4m)\times \left(1+\frac{D_{i}^{*}(8m)}{D_{i}^{*}(4m)}\right)=D_{i}(4m)\times (1+K_{i}) \end{array}$$ (4)

where *K*_i_ is the ratio of the 8-month dose to the first 4-month dose in terms of ambient dose equivalent measured at the reference site of *i*^th^ municipality.

The ratio *K*_i_ was expected to differ by place. First, *K*_i_ was expected to depend on the concentration ratio of short-lived radionuclides (such as ^132^Te/^132^I and ^131^I) to long-lived radionuclides (such as ^137^Cs and ^134^Cs) in the environment. That is, areas where radioactive plumes with higher concentrations of short-lived radionuclides passed were expected to have a smaller *K*_i_ due to a larger contribution of the first 4-month dose. Second, *K*_i_ was expected to depend on ground conditions at measurement places; for example, some areas have a winter snow cover, known to lead to a reduced ambient dose rate.^23^ Thus, *K*_i_ was estimated for each municipality based on available ambient dose rate data described in the next section.

### Available ambient dose rate data

**Figure 4.**
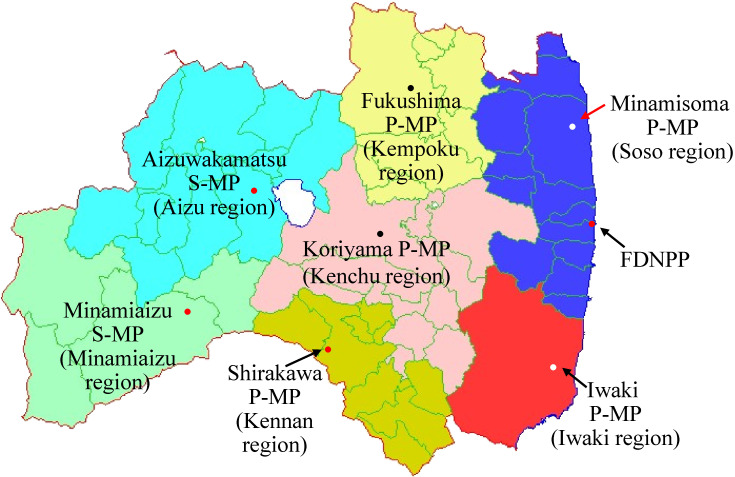
Locations of MPs in seven regions of Fukushima Prefecture. As shown in Figure 1, two types of MPs were used. MPs for Aizuwakamatsu and Minamiaizu were the stationary-type MP (S-MP) which measured absorbed dose in air, while the other MPs were the portable-type MP (P-MP) which measured ambient dose equivalent.

**Figure 5.**
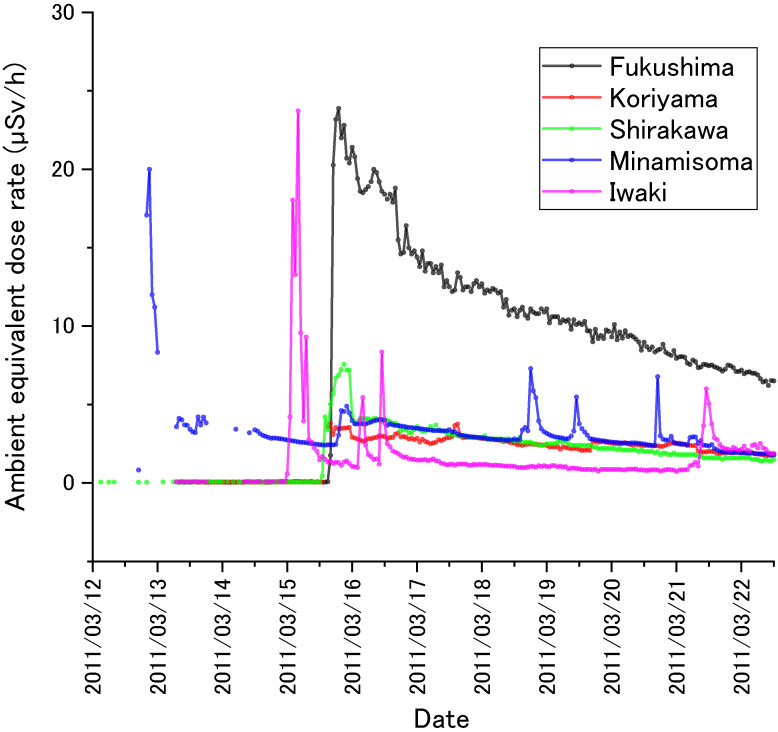
Changes in ambient equivalent dose rate in an early stage after the accident. Data reported from the five S-MPs were plotted.

There were limited numbers of stations which had monitored ambient dose rate in Fukushima Prefecture before the accident. The map of Figure 4 shows seven regions of Fukushima Prefecture; each had a station (hereafter, monitoring post [MP]). Five MPs were portable-type MPs (P-MPs) and two were stationary-type MPs (S-MPs) (compare to Figure 1).^24^ Figure 5 plots reported data^25^ from the P-MPs. The MPs continuously measured ambient dose rate every hour before and after March 12, 2011, although data during several hours were lacking at some stations due to earthquake damage (eg, Minamisoma MP). Corresponding to the times of the radioactive plumes, ambient dose rate for each MP jumped up.

Similar to equation (3), the following relationship was assumed for the S-MPs which measured absorbed dose rate in air (Figure 4).

$$D_{i}(t)=D_{i}^{\prime \prime }(t)\times F_{2}\times R_{i}$$ (5)

Here $D_{i}^{\prime \prime }(t)$ is the absorbed dose in air measured with the S-MP located in *i*^th^ municipality for period *t*, *F*_2_ is the conversion factor from absorbed dose rate in air to effective dose (Figure 2).

**Figure 6.**
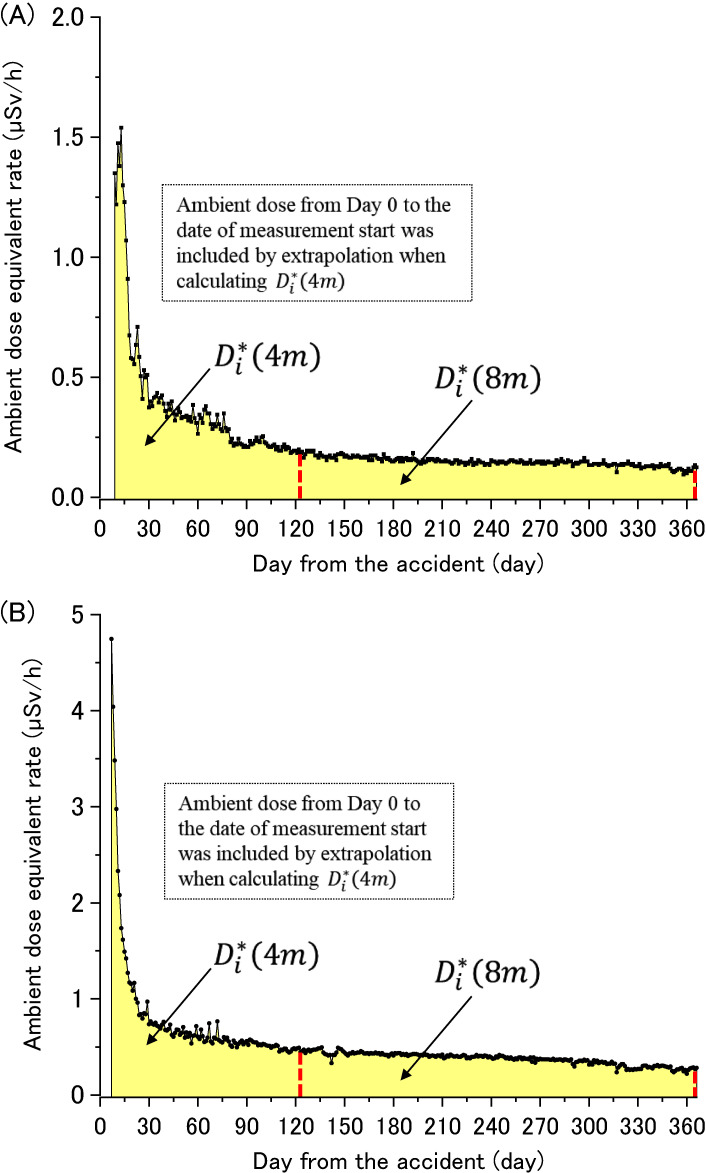
Changes in ambient dose equivalent measured with SVs in the first year. (**A**) Soma City Office in Soso region and (**B**) Kunimi Town Office in Kempoku region. Background dose was subtracted. $D_{i}^{*}(4m)$ and $D_{i}^{*}(8m)$ were estimated based on yellow shaded areas. Figure 7 shows locations of the municipalities.

Several days after the accident, measurements of ambient dose equivalent at designated places were begun using survey meters (SVs). In most cases, these measurements were continued in the first year. At least one place from each non-evacuated area municipality was selected; typically, municipality head or branch offices. As two examples, reported ambient dose equivalent rates at Soma City Office and Kunimi Town Office are shown in Figure 6A and Figure 6B, respectively.

The start date of SV measurements differed from place to place (March 17 to 21, 2011). Since it took at least several days to begin SV measurements after March 12, 2011, the ambient dose equivalent from March 12 to the start date of the SV measurements was not available.

Thus, the 4-month ambient dose equivalent for each municipality, $D_{i}^{*}(4m)$ in equation (4), was estimated using the measurement results for MPs which continued to measure ambient dose.

$$\begin{array}{rl} D_{i}^{*}(4m) & =D_{i}^{*}(T)\times \frac{D_{R}^{\prime \prime }(4m)}{D_{R}^{\prime \prime }(T)}\\ & =(D_{Mi}^{*}(T)-D_{Bi}^{*}(T))\times \frac{\left(D_{\text{MR}}^{\prime \prime }(4m)-D_{\text{BR}}^{\prime \prime }(4m)\right)}{\left(D_{\text{MR}}^{\prime \prime }(T)-D_{\text{BR}}^{\prime \prime }(T)\right)} \end{array}$$ (6)

$$\begin{array}{rl} D_{i}^{*}(4m) & =D_{i}^{*}(T)\times \frac{D_{R}^{*}(4m)}{D_{R}^{*}(T)}\\ & =(D_{Mi}^{*}(T)-D_{Bi}^{*}(T))\times \frac{\left(D_{\text{MR}}^{*}(4m)-D_{\text{BR}}^{*}(4m)\right)}{\left(D_{\text{MR}}^{*}(T)-D_{\text{BR}}^{*}(T)\right)} \end{array}$$ (7)

Here *T* is the measurement period for a SV (<4 months), $D_{R}^{\prime \prime }$ (or $D_{R}^{*}$ ) is the ambient dose measured with the MP of region *R* where the targeted *i*^th^ municipality is located (Figure 4). Table 1 lists variables used in the equation.

Andoh et al^26^ estimated municipality-specific background doses (ambient dose equivalent) due to terrestrial radiation (0.034 to 0.072 µSv/h) from car-borne surveys conducted across a wide area of Fukushima Prefecture. $D_{Bi}^{*}(T)$ was calculated from these values. The ambient dose rate for each MP measured before the accident was used to calculate background doses ( $D_{BR}^{\prime \prime }(T)$ , $D_{BR}^{*}(T)$ , $D_{BR}^{\prime \prime }(4m)$ and $D_{BR}^{*}(4m)$ ).

Similarly, $D_{i}^{*}(8m)$ was calculated, which gives a *K*_i_ value and the first-year effective dose for each municipality by equation (4). In the case of municipalities where the MPs were located, $D_{i}^{\prime \prime }(4m)$ and $D_{i}^{\prime \prime }(8m)$ (or $D_{i}^{*}(4m)$ and $D_{i}^{*}(8m)$ ) were calculated from MP data, even if SV data were available. After estimating $D_{i}^{*}(4m)$ (or $D_{i}^{\prime \prime }(4m)$ ), *R*_i_ was estimated for each municipality by equation (3) or (5).

### Comparison with other available data

As a validation of above-mentioned method, the doses estimated by the method were compared with other available data in the following way.

First, a comparison was made with the first-year doses estimated in the UNSCEAR 2013 report.^1^ Another comparison was made with data from personal dosimeter measurements. Around a half-year after the accident, measurements of external doses for residents using integrating-type personal dosimeters were started on a large scale by local governments of Fukushima Prefecture. Typical measurement periods for personal dosimeters were a few months.

Personal doses (Figure 1 and Figure 2) obtained in the geometrical conditions of the affected areas in Fukushima Prefecture are known to be comparable with the effective dose.^21^ Thus, $D_{i}^{p}(t_{p})$ , which is the average additional dose estimated with personal dosimeters for a period (*t*_p_) at *i*^th^ municipality can be compared with additional effective dose estimated for the same period by a similar method to equations (3) and (5) (Figure 3).

$$D_{i}(t_{p})=D_{i}^{*}(t_{p})\times F_{1}\times R_{i}$$ (8)

However, when comparing $D_{i}^{p}(t_{p})$ with *D_i_*(*t_p_*), background dose should be treated cautiously. In Fukushima Prefecture, integrating-type personal dosimeters from two manufacturers were mainly used.^27^ One manufacturer seemed to use 0.54 mSv per year as the background dose, which was based on measured values in a place distant from Fukushima Prefecture. The other used “control badges” to estimate background dose. Even in the case that “additional doses” subtracting these background doses are disclosed, the estimated background doses are not necessarily disclosed for both types of dosimeters. Thus, disclosed “additional dose” could be affected by the estimation of background dose.

Then, the comparison was made for doses including background dose. For personal dose, the following was used:

$$D_{Mi}^{p}(t_{p})=D_{i}^{p}(t_{p})+D_{Bi}^{p}(t_{p})$$ (9)

where $D_{Bi}^{p}(t_{p})$ is the estimated background dose for a period *t*_p_.

Thus, data on personal dosimeter measurements which meet the following conditions were selected: (1) the background dose $D_{Bi}^{p}$ is disclosed in addition to average additional dose; (2) the measurement period for personal dosimeters is disclosed; and (3) the period is included in the first year after the accident start date (until March 11, 2012). In total, seven datasets from four municipalities were available.^28^^–^^31^

On the other hand, average effective dose due to external radiation including background for *i*^th^ municipality, which should be compared with $D_{Mi}^{p}(t_{p})$ , can be calculated using the results for P-MPs or SVs:

$$\begin{array}{rl} D_{i}(t_{p})+D_{Bi}(t_{p})+D_{\text{UB}}(t_{p}) & =(D_{Mi}^{*}(t_{p})-D_{Bi}^{*}(t_{p}))\times F_{1}\times R_{i}\\ & \;+D_{Bi}^{*}(t_{p})\times F_{1}+D_{\text{UB}}(t_{p}) \end{array}$$ (10)

Table 1 shows the variables.

Because personal dosimeters are sensitive to cosmic rays as well as terrestrial radiation, dose from cosmic rays should be considered for comparison (Figure 2). The effective dose from cosmic rays, *D*_UB_(*t*_p_), was assumed to be the same for all municipalities and calculated according to the measurement period (*t*_p_) on the basis of an annual dose of 0.21 mSv.^32^

### Ethics statement

The study protocol of the Basic Survey was reviewed and approved by the Ethics Review Committee of Fukushima Medical University (Nos. 1257, 1275, 1294).

## RESULTS AND DISCUSSION

### First 4-month doses based on the Basic Survey

**Figure 7.**
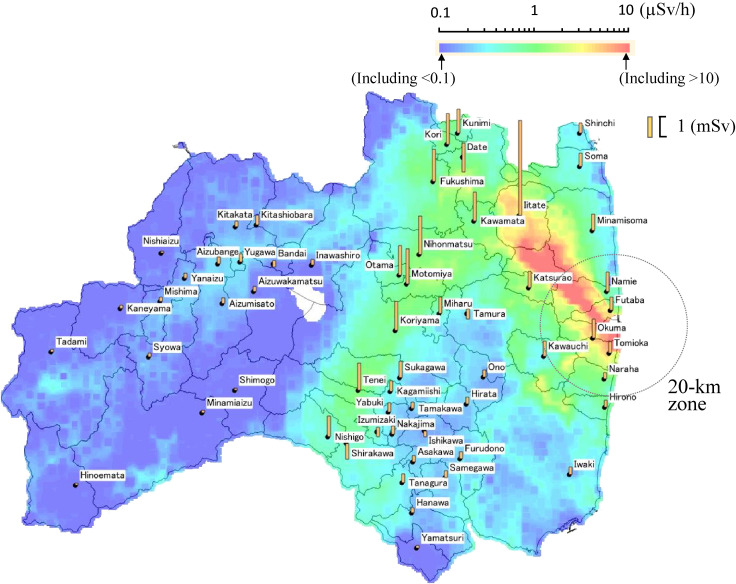
Ambient dose equivalent rate map of Fukushima Prefecture and the first 4-month average individual doses (bar graphs) classified by residential places at the time of accident. Decay correction for ambient dose equivalent rate was made to May 31, 2012. Ambient dose equivalent rate data were taken from: https://emdb.jaea.go.jp/emdb/

Arithmetic mean, standard deviation, median, 90^th^ and 95^th^ percentile values for the first 4-month doses for each municipality are tabulated in Table 2. Arithmetic means of the 4-month doses are shown as bar graphs in Figure 7, together with an ambient dose equivalent rate map.

**Table 2.** Arithmetic mean, median, 90^th^ and 95^th^ percentile values of the first 4-month doses for each municipality

Region	Municipality	Number of respondents	First 4-month dose (mSv)				

			Arithmetic mean	Standard deviation	Median	90th percentile	95th percentile
Kempoku	Fukushima	78,379	1.37	0.52	1.4	2	2.2
	Nihonmatsu	13,605	1.64	0.52	1.7	2.2	2.4
	Date	14,774	1.25	0.55	1.2	1.9	2.2
	Motomiya	7,493	1.51	0.49	1.5	2.1	2.3
	Koori	3,136	1.32	0.33	1.3	1.7	1.8
	Kunimi	2,415	1.03	0.31	1	1.4	1.5
	Kawamata	3,664	1.24	0.60	1.1	1.8	2.3
	Otama	1,602	1.27	0.46	1.2	1.9	2.1

Kenchu	Koriyama	73,110	1.25	0.61	1.3	2	2.3
	Sukagawa	14,422	0.71	0.50	0.5	1.5	1.7
	Tamura	8,395	0.44	0.34	0.3	0.9	1.2
	Kagamiishi	2,445	0.46	0.21	0.4	0.7	0.8
	Tenei	1,052	1.16	0.53	1.2	1.8	2
	Ishikawa	3,237	0.26	0.17	0.2	0.4	0.5
	Tamakawa	1,205	0.28	0.21	0.2	0.4	0.6
	Hirata	1,335	0.31	0.22	0.3	0.5	0.7
	Asakawa	1,247	0.28	0.14	0.3	0.4	0.5
	Furudono	1,089	0.31	0.19	0.3	0.4	0.6
	Miharu	3,970	0.71	0.37	0.6	1.1	1.3
	Ono	2,111	0.31	0.26	0.2	0.6	0.8

Kennan	Shirakawa	13,774	0.67	0.24	0.7	0.9	1.1
	Nishigo	4,287	0.89	0.28	0.9	1.2	1.3
	Izumizaki	1,185	0.42	0.18	0.4	0.6	0.7
	Nakajima	857	0.36	0.18	0.3	0.5	0.6
	Yabuki	3,460	0.41	0.20	0.4	0.6	0.8
	Tanagura	2,586	0.38	0.18	0.4	0.5	0.6
	Yamatsuri	1,165	0.12	0.14	0.1	0.2	0.3
	Hanawa	1,892	0.23	0.15	0.2	0.3	0.4
	Samegawa	664	0.31	0.18	0.3	0.4	0.6

Aizu	Aizuwakamatsu	23,944	0.23	0.14	0.2	0.3	0.3
	Kitakata	9,000	0.26	0.14	0.2	0.4	0.4
	Kitashiobara	483	0.40	0.15	0.4	0.5	0.6
	Nishiaizu	1,018	0.09	0.09	0.05	0.2	0.2
	Bandai	666	0.29	0.16	0.3	0.4	0.4
	Inawashiro	2,895	0.24	0.17	0.2	0.4	0.6
	Aizubange	2,664	0.30	0.12	0.3	0.4	0.4
	Yugawa	601	0.35	0.13	0.3	0.4	0.5
	Yanaizu	559	0.22	0.16	0.2	0.3	0.3
	Mishima	247	0.19	0.07	0.2	0.2	0.3
	Kaneyama	409	0.14	0.14	0.1	0.2	0.3
	Syowa	246	0.17	0.14	0.2	0.2	0.2
	Aizumisato	3,659	0.25	0.14	0.2	0.3	0.4

Minamiaizu	Shimogo	974	0.08	0.12	0.05	0.1	0.2
	Hinoemata	103	0.08	0.08	0.05	0.1	0.2
	Tadami	887	0.13	0.11	0.1	0.2	0.2
	Minamiaizu	3,052	0.11	0.14	0.05	0.2	0.2

Soso	Soma	10,610	0.58	0.34	0.6	0.8	1
	Minamisoma	26,025	0.70	0.56	0.6	1.4	1.7
	Hirono	1,902	0.30	0.36	0.2	0.6	0.8
	Naraha	3,551	0.28	0.34	0.2	0.6	0.9
	Tomioka	7,067	0.49	0.58	0.3	1.2	1.5
	Kawauchi	1,333	0.65	0.58	0.6	1.3	1.5
	Okuma	4,811	0.80	0.79	0.7	1.4	1.7
	Futaba	3,265	0.56	0.89	0.3	1.3	1.7
	Namie	8,471	0.85	1.22	0.6	1.7	2.2
	Katsurao	693	0.71	0.61	0.5	1.5	1.9
	Shinchi	2,200	0.45	0.17	0.5	0.6	0.7
	Iitate	2,338	4.03	2.43	3.8	7	8.4

Iwaki	Iwaki	74,139	0.33	0.18	0.3	0.5	0.6

Total		466,368	—	—	—	—	—

As shown in Figure 7, the first 4-month doses for municipalities in the 20-km zone were relatively small, considering the ambient dose equivalent rate levels. The highest dose was found for Iitate Village, outside the 20-km zone from the FDNPP. This was because (1) evacuation orders were given soon for the 20-km zone on March 12, 2011, and (2) most people followed them and moved outside the zone,^33^ which was effective in reducing external dose in an early stage.

On April 22, 2011, a Deliberate Evacuation Area, located to the northwest of the FDNPP, was established by the national government beyond the 20-km evacuation zone to include areas where the projected dose criterion of 20 mSv in 1 year might be exceeded; however, relocation of people from this area was not implemented for approximately 1 month. As a result, evacuation was delayed for the Deliberate Evacuation Area compared with the 20-km zone and Iitate Village, in the area, had the highest dose. Still, its average 4-month dose was 4.0 mSv.

### Ambient dose data for the first year by municipality

The calculated results for *K*_i_ are shown in Table 3. For three cities (Date, Motomiya, and Sukagawa), consecutive ambient dose equivalent rate data for designated points were not available throughout the first year. Thus, an average *K*_i_ for municipalities belonging to Kempoku region (excluding Date and Motomiya Cities) was applied for Date and Motomiya Cities. The footnote of Table 3 explains the situation for Sukagawa City.

**Table 3.** First-year municipality-average doses estimated by UNSCEAR and the present study, together with *R*_i_ and *K*_i_ values

Region	Municipality	Number of respondents	*R*_i_	*K*_i_	Municipality-average dose (mSv)			Ratio

					4-month dose	1-y dose (this study)	1-y dose (UNSCEAR)	
Kempoku	Fukushima	78,379	0.355	0.817	1.37	2.49	3.02	0.82
	Nihonmatsu	13,605	0.945	0.979	1.64	3.25	2.44	1.33
	Date^a^	14,774	0.463	0.927	1.25	2.42	1.95	1.24
	Motomiya^a^	7,493	0.581	0.927	1.51	2.91	1.52	1.91
	Kori	3,136	0.515	1.016	1.32	2.66	2.65	1.00
	Kunimi	2,415	0.662	0.851	1.03	1.90	1.14	1.67
	Kawamata	3,664	0.668	1.055	1.24	2.54	1.23	2.07
	Otama	1,602	0.724	0.843	1.27	2.34	2.11	1.11

Kenchu	Koriyama	73,110	0.389	0.821	1.25	2.28	2.01	1.14
	Sukagawa^b^	14,422	0.234	0.929	0.71	1.36	1	1.36
	Tamura	8,395	1.193	0.940	0.44	0.85	0.52	1.63
	Kagamiishi	2,445	0.851	0.897	0.46	0.88	0.74	1.19
	Tenei	1,052	0.503	0.955	1.16	2.27	1.39	1.63
	Ishikawa	3,237	1.361	0.815	0.26	0.47	0.15	3.12
	Tamakawa	1,205	1.009	1.012	0.28	0.57	0.2	2.84
	Hirata	1,335	1.161	1.115	0.31	0.67	0.28	2.38
	Asakawa	1,247	0.937	1.014	0.28	0.57	0.3	1.91
	Furudono	1,089	1.057	1.089	0.31	0.65	0.28	2.31
	Miharu	3,970	0.976	0.986	0.71	1.41	1.1	1.28
	Ono	2,111	1.510	0.791	0.31	0.55	0.29	1.89

Kennan	Shirakawa	13,774	0.555	1.026	0.67	1.35	0.98	1.38
	Nishigo	4,287	0.607	1.163	0.89	1.93	1.15	1.68
	Izumizaki	1,185	0.334	1.161	0.42	0.90	0.75	1.20
	Nakajima	857	0.991	1.021	0.36	0.72	0.32	2.26
	Yabuki	3,460	0.587	1.028	0.41	0.83	0.43	1.92
	Tanagura	2,586	0.575	0.898	0.38	0.73	0.58	1.25
	Yamatsuri	1,165	0.953	0.991	0.12	0.23	0.08	2.92
	Hanawa	1,892	0.605	1.122	0.23	0.49	0.25	1.97
	Samegawa	664	1.104	0.975	0.31	0.62	0.29	2.13

Aizu	Aizuwakamatsu	23,944	0.680	1.048	0.23	0.46	0.33	1.40
	Kitakata	9,000	1.331	1.027	0.26	0.52	0.33	1.58
	Kitashiobara	483	1.294	0.952	0.40	0.78	0.64	1.22
	Nishiaizu	1,018	1.144	0.535	0.09	0.14	0.07	2.02
	Bandai	666	0.814	0.995	0.29	0.57	0.27	2.12
	Inawashiro	2,895	1.024	1.094	0.24	0.51	0.32	1.59
	Aizubange	2,664	0.954	0.925	0.30	0.58	0.52	1.11
	Yugawa	601	0.749	0.705	0.35	0.60	0.49	1.23
	Yanaizu	559	0.913	0.860	0.22	0.41	0.17	2.39
	Mishima	247	1.300	0.971	0.19	0.37	0.17	2.19
	Kaneyama	409	1.242	0.989	0.14	0.28	0.03	9.36
	Syowa	246	1.010	0.884	0.17	0.32	0.16	2.01
	Aizumisato	3,659	1.216	0.895	0.25	0.48	0.19	2.53

Minamiaizu	Shimogo	974	1.249	0.977	0.08	0.17	0.04	4.13
	Hinoemata	103	1.565	0.713	0.08	0.14	0.03	4.52
	Tadami	887	2.121	0.569	0.13	0.21	0.14	1.48
	Minamiaizu	3,052	0.981	1.720	0.11	0.30	0.07	4.23

Soso	Soma	10,610	0.612	0.556	0.58	0.90	0.69	1.30
	Shinchi	2,200	0.817	0.845	0.45	0.83	0.7	1.19

Iwaki	Iwaki	74,139	0.495	0.646	0.33	0.54	0.78	0.69

Total		466,368	—	—	—	—	—	—

^a^SV measurements for these city offices started in March 2011, but stopped in the middle of June. The available data were extrapolated to the 4-month dose using data from Fukushima MP of Kempoku region where these cities are located. Then, values of *R*_i_ were estimated.

^b^SV measurements for Sukagawa City Office started in March 2011 but stopped at the beginning of September. Those for a branch office of Sukagawa City started in July and continued until March 2012. The first 4-month ambient dose equivalent at the branch office was estimated using (1) that estimated for the City Office and (2) a ratio deduced from a comparison between data on both sites in July and August 2011. Then, *K*_i_ and *R*_i_ values were estimated.

The *K*_i_ values were compared with that estimated by another method in the following way. Yoshimura et al^34^ deduced an equation which approximates changes in additional ambient dose equivalent rate in the first year as follows:

$$\text{normAD}(t)=a_{0}\times \exp(-k_{1}\times t)+b_{0}\times \exp(-k_{2}\times t)$$ (11)

where *normAD*(*t*) is the normalized ambient dose equivalent rate (background subtracted), *a*_0_ = 6.2 × 10^−5^, *k*_1_ = 72, *b*_0_ = 6.9 × 10^−6^, *k*_2_ = 1.05 for places of residential areas with paved surfaces. Most of the places (seven among 11 points) were located in the northwestern direction of FDNPP.

Using this equation, the ratio *K* can be calculated as follows.

$$K=\frac{\int_{t=123}^{t=366}\text{normAD}(t)}{\int_{t=0}^{t=122}\text{normAD}(t)}$$ (12)

The calculated *K* was 0.845, which was similar to the average *K*_i_ (0.927) for municipalities of Kempoku region (excluding Date and Motomiya Cities) located in the northwestern direction of FDNPP.

The *K*_i_ values for Soma and Iwaki Cities were smaller than those in other municipalities. This would be because plumes with rich short-lived radionuclides were directed to these areas on March 12 and 15, 2011,^35^ which increased the first 4-month dose (see Figure 6A in comparison with Figure 6B).

The *K*_i_ values for Minamiaizu region had large variation. Since additional dose was the lowest in this region, uncertainty of background dose estimation affected the estimation of *K*_i_ values.

### External doses estimated by extrapolation and their comparison with other data

**Figure 8.**
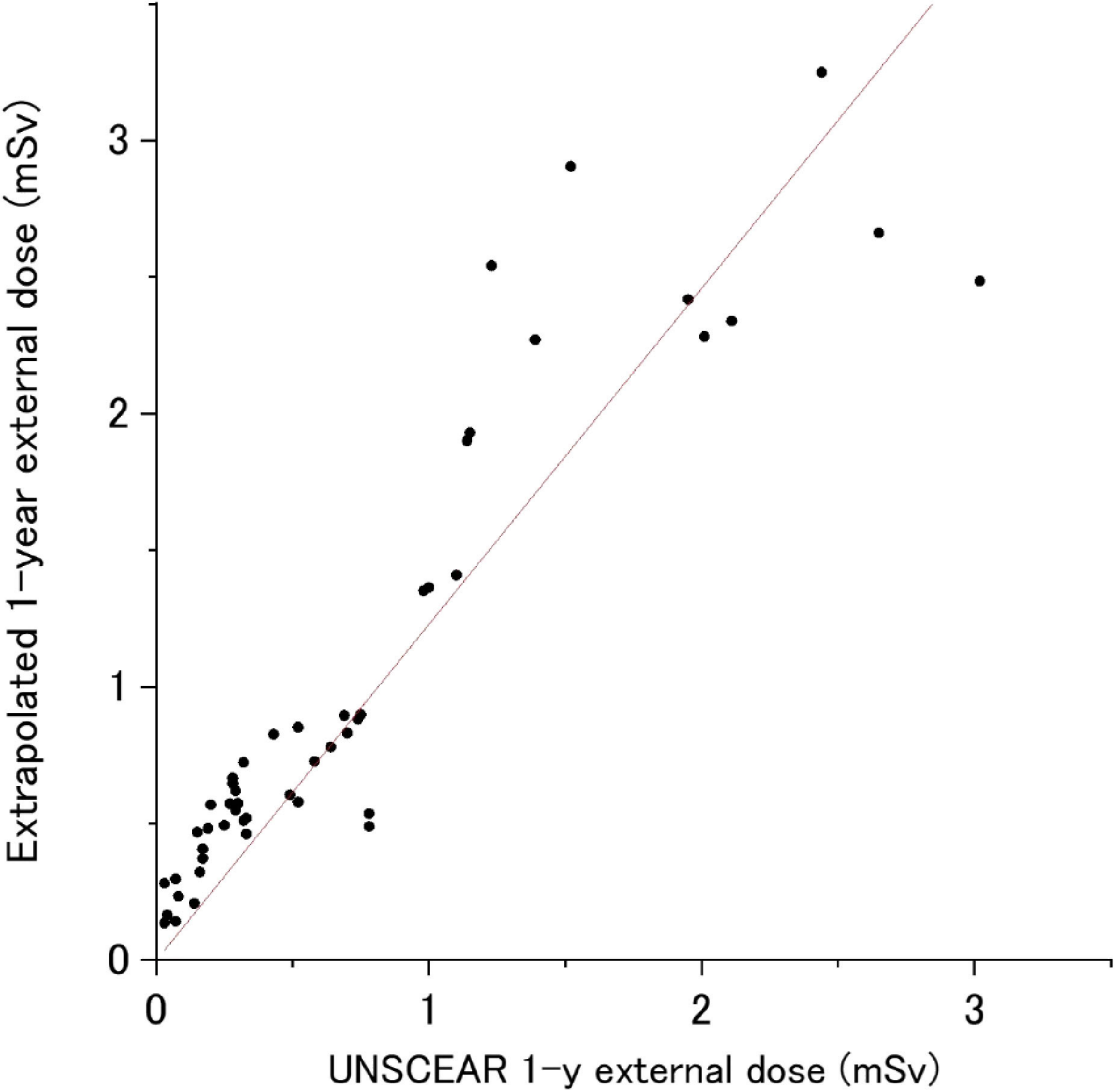
Comparison of first-year doses estimated by UNSCEAR and extrapolated doses by the present study

The first-year extrapolated doses for non-evacuated areas were compared with the UNSCEAR estimation, as shown in Figure 8 and Table 3. Although some parts of Tamura City and Kawamata Town were designated as evacuation areas, the population of the evacuation areas was around one tenth of the total municipality population for both municipalities.^1^ Thus, the first-year doses were also calculated for these municipalities. Since most of Soso region was designated as evacuation areas, the first-year dose was calculated for only two municipalities.

While there was a good linear correlation between them (coefficient of determination: 0.92), the estimated doses in the present study were generally higher (slope of the regression line: 1.23). The estimated first-year doses were based on available actual data (people’s behaviors in the first 4 months and measured ambient dose rates in the first year), while the UNSCEAR estimation was based on measurement data of radionuclide deposition, assumed behavior patterns and model prediction of radionuclide removal (horizontal migration due to wash-off; ie, weathering), as well as radioactive decay. It was reported that actual removal rate of radioactive cesium by weathering was slower than the UNSCEAR estimation,^34^ which could give larger doses due to longer residential time of radioactive cesium in the present study.

The radiation reduction factor *R*_i_ for each municipality is also tabulated in Table 3. The values sometimes exceeded 1. According to the original idea of *R* (equation [1]), it should be less than 1. However, the value of *R*_i_ depends on location of the reference site where the ambient dose rate was measured. If the ambient dose rate at the reference site was generally lower than that for population-concentrated areas (Figure 3), it is possible that the value of *R*_i_ exceeded 1.

Table 4A and Table 4B show results for municipality-average dose estimation from personal dosimeter measurements and ambient doses, respectively. The difference was from −26% to −1% (average: −10%). Although the comparison was made only for seven datasets from four different municipalities, there was reasonable agreement between them.

**Table 4A.** Results for personal dose measurements selected for comparison

No	Municipality	Ref No.	Start of measurement	End of measurement	Period (days)	Number of participants	Main target population	Average additional dose	Background dose during the period	Total average dose during the period

								a	b	c = a + b
1	Fukushima^a^	28	Jan 18, 2011	Feb 25, 2012	30	125	Adults	—	—	0.138
2	Koriyama	29	Oct 5, 2011	Nov 6, 2011	33	25,551	Children	0.12	0.06	0.180
3	Koriyama	29	Nov 7, 2011	Jan 9, 2012	64	24,115	Children	0.17	0.12	0.290
4	Koriyama	29	Jan 10, 2012	Feb 29, 2012	51	22,287	Children	0.13	0.09	0.220
5	Sukagawa	30	Sep 6, 2011	Nov 7, 2011	63	11,461	Children, pregnant women	0.15	0.1	0.250
6	Sukagawa	30	Nov 7, 2011	Feb 7, 2012	93	11,446	Children, pregnant women	0.18	0.15	0.330
7	Yabuki	31	Oct 1, 2011	Dec 22, 2011	83	1,484	Children	0.07	0.123	0.193

^a^According to Ref. 28, a fixed measurement period of 30 days was selected between Jan 18 and Feb 25, 2012.

**Table 4B.** Municipality-average effective doses estimated from ambient dose equivalent and *R*_i_ values for periods corresponding to Table 4A

No	Municipality	Data source	Ambient dose equivalent at a reference site in targeting municipality for a period corresponding to personal dosimeter measurement (mSv)			Effective dose for the population of the targeted municipality for a period corresponding to personal dosimeter measurement (mSv)				Difference with PD

			Measured dose	Estimated background dose due to terrestrial radiation	Additional dose	Average additional dose estimated using *R*_i_	Background dose from terrestrial radiation	Background dose from universe	Total average effective dose (mSv)	

			a	b	c = a − b	d = c × *F*_1_ × *R*_i_	e = b × *F*_1_	f	g = d + e + f	h = c in Table 4A/g
1	Fukushima^a^	P-MP	0.524	0.029	0.495	0.105	0.017	0.017	0.140	0.99
2	Koriyama	P-MP	0.659	0.040	0.619	0.144	0.024	0.019	0.187	0.96
3		P-MP	1.165	0.077	1.088	0.254	0.046	0.037	0.337	0.86
4		P-MP	0.787	0.061	0.725	0.169	0.037	0.029	0.235	0.94
5	Sukagawa	SV	1.364	0.095	1.268	0.178	0.057	0.036	0.272	0.92
6		SV	1.793	0.141	1.653	0.232	0.084	0.054	0.370	0.89
7	Yabuki	SV	0.519	0.120	0.399	0.141	0.072	0.048	0.260	0.74

^a^Measured dose for Fukushima MP was integrated from Jan 22 to Feb 20 (30 days) in 2012, although a period of 30 days was selected between Jan 18 and Feb 25, 2012 for personal dosimeter measurements (see footnotes of Table 4(a)).

### Limitations

There are several limitations in the present study. First, the radiation reduction factor for population of each municipality was assumed to be the same between the first 4 months and the subsequent 8 months. However, people’s daily time budget might change over time after the accident. Second, the population was assumed to continue to live in the same living environments with ambient dose rates decreasing at the same trend as the reference site. However, some people left their residence towns even in non-evacuated areas. Also, the decreasing trend of ambient dose might differ even within the same municipality. Third, some measurement errors could be associated with MP and SV measurements of ambient dose. Fourth, it was not certain whether participants for personal dosimeter measurements were representative of the whole population of a targeted municipality or not.

### Conclusion

The first 4-month dose for each municipality was presented based on the Basic Survey results. Next, data on ambient dose rates reported within the first year after the accident were analyzed to extrapolate the 4-month doses to the first-year dose for municipalities of non-evacuated areas. The first-year extrapolated dose was compared with UNSCEAR estimation. Generally, doses determined in the present study were higher, which may be due to slower weathering of cesium than the UNSCEAR estimation assumed. The extrapolated doses were also compared with doses measured by personal dosimeters. They were in reasonable agreement (within 30%), suggesting that the extrapolation was reasonable. The estimated doses will be the basis for future epidemiological studies related to the Fukushima Health Management Survey.
